# Ultrasound-guided radiofrequency ablation for recurrent contralateral tumors that developed after initial surgery for papillary thyroid carcinoma

**DOI:** 10.3389/fendo.2026.1782098

**Published:** 2026-05-07

**Authors:** Shaowei Xue, Xiaoshi Bao, Ziyu Jiao, Siming Chen, Longjun Guo

**Affiliations:** 1 Rehabilitation Ultrasonics Department, Beijing Rehabilitation Hospital Affiliated to Capital Medical University, Beijing, China; 2 Department of Ultrasound, First Medical Center, Chinese PLA General Hospital, Beijing, China

**Keywords:** interventional ultrasound, papillary thyroid carcinoma, radiofrequency ablation, thyroid, ultrasound

## Abstract

**Objective:**

The aim of this study was to investigate the efficacy and safety of ultrasound-guided radiofrequency ablation (RFA) for recurrent contralateral tumors that developed after initial surgery for papillary thyroid carcinoma (PTC).

**Methods:**

This retrospective study included 32 postoperative patients with PTC with unifocal recurrent contralateral tumors who underwent RFA between January 2015 and December 2021. The RFA procedures were performed using the hydrodissection technique, moving-shot technique, and enlarged ablation technique. Patients were followed up at 1, 3, 6, 12 months, and every 12 months thereafter. Local tumor progression (LTP), volume reduction rate (VRR), tumor complete disappearance, complications, and delayed surgery were evaluated during the follow-up.

**Results:**

The mean follow-up period of RFA was 54.4 ± 24.3 months. Two patients developed lymph node metastases (LNMs), and the overall incidence of LTP was 6.3% (2/32). No newly identified tumors or distant metastases were observed. The median VRR was 100%, and 90.4% of the tumors had completely disappeared. One patient experienced transient voice change (3.1%). Because of anxiety, one patient underwent delayed surgery (3.1%), and no residual cancer cells, occult tumors, or cervical LNMs were found. No patients experienced hypothyroidism following RFA.

**Conclusions:**

RFA may be considered as a feasible and safe local treatment for recurrent contralateral tumors after the initial surgery for PTC in select patients who are not candidates for or who refuse repeat surgery.

## Introduction

Thyroid cancer is the most common malignancy of the endocrine system and ranks seventh in incidence among all cancers (1). Papillary thyroid carcinoma (PTC) is the most common subtype of thyroid cancer, and the majority of PTC cases are low risk with an excellent prognosis. Although total thyroidectomy followed by adjuvant radioactive iodine (RAI) therapy is recommended for PTC, more lines of evidence have demonstrated that thyroid lobectomy alone achieves comparable survival outcomes in patients with low-risk PTC (2). Therefore, the American Thyroid Association (ATA) Guidelines recommend that lobectomy may be sufficient for intrathyroidal PTC [stage T1 or T2 without clinically involved lymph node metastasis (LNM)] (3, 4). However, loco-regional recurrence can occur in the cervical LNs and the remnant contralateral lobe following lobectomy. Repeat surgery is the standard treatment. However, it may not be suitable for all the patients due to surgical contraindications or patient preferences.

Ultrasound (US)-guided radiofrequency ablation (RFA) and other thermal ablation techniques have been recommended as effective and safe alternatives to surgery for benign thyroid nodules and recurrent thyroid cancers (4–11). Many studies have reported favorable outcomes of ablation in treating cervical LNM (12–15). The pooled proportion of volume reduction rate (VRR) ranged from 88.4% to 93%, with a significant decrease in thyroglobulin levels (16, 17). However, information on the outcomes of RFA for recurrent contralateral tumors that developed after the initial surgery for PTC remains scarce.

Therefore, the purpose of this study was to evaluate the clinical outcomes of RFA for recurrent contralateral tumors that developed after initial surgery for PTC.

## Materials and methods

### Patients

This study was approved by the Institutional Review Board of our institution, and the requirement for obtaining informed consent from patients was waived because of its retrospective nature. All the patients provided written information consent before RFA. The inclusion criteria were as follows: (1) patients who previously underwent thyroid lobectomy with concomitant central neck dissection for PTC treatment; (2) those who presented with a newly found tumor in the contralateral lobe after initial surgery, and confirmed as PTC by fine-needle aspiration (FNA) or core-needle biopsy (CNB); (3) the maximum diameter is ≤10.0 mm; (4) no clinical or imaging evidence of extrathyroidal invasion, LNM, and distant metastasis on US and chest computed tomography (CT); (5) patients who were unsuitable for repeat surgery or rejected repeat surgery clearly; and (6) follow-up period was ≥12 months. The exclusion criteria were as follows: (1) conscious disturbance or neck extension disorder that could not tolerate ablation procedure; (2) serious organ failure; and (3) follow-up period < 12 months or incomplete data.

**Figure 1:**
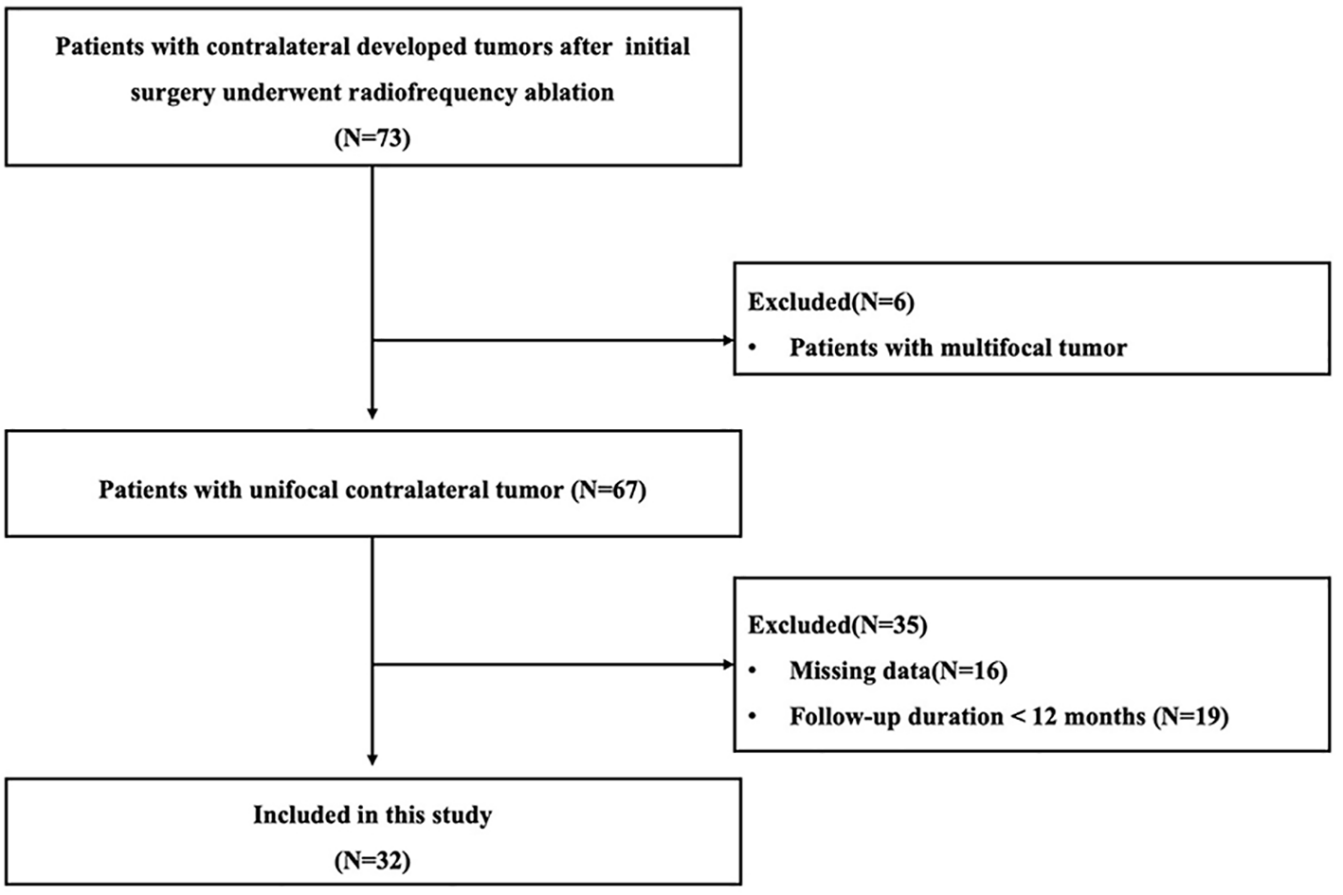
Study flow diagram.

The electronic medical records of 73 consecutive postoperative ATA low-risk patients with contralateral tumors who received RFA between January 2015 and December 2021 were reviewed. Among them, patients with multifocal tumor (*n* = 6) or incomplete data (*n* = 16) or follow-up time < 12 months (*n* = 19) were excluded. The remaining 32 patients with unifocal contralateral tumor were included in this study (**Figure 1**). Among them, 21 patients rejected repeat surgery clearly because of cosmetic reason or concerns of complications. The other 11 patients were unsuitable for repeat surgery (renal insufficiency, *n* = 5; breast cancer, *n* = 1, poor pulmonary function, *n* = 2; hypertension, *n* = 3). The comparison between included and excluded patients are shown in Supplement 1. Because patients only underwent thyroid lobectomy with concomitant central neck lymph node dissection, no tumor was found in the contralateral lobe during the initial treatment. Therefore, these newly found contralateral tumors were considered as local recurrences by a multidisciplinary team in the postoperative follow-up examination. Moreover, no postoperative TSH-suppressive therapy was performed after the initial surgery.

### Pre-ablation evaluation

All patients underwent laboratory tests, US examination, and chest CT. The laboratory tests included complete blood count, thyroid function tests, and coagulation tests. The thyroid function tests included free triiodothyronine (fT3; normal range, 2.76–6.30 pmol/L), free thyroxine (fT4; normal range, 10.42–24.32 pmol/L), and thyroid-stimulating hormone (TSH; normal range, 0.23–5.50 mU/L). US was performed to evaluate the tumor, thyroid capsule, and cervical LN. The volume of tumor was calculated using the following equation: *V* = π*abc*/6 (*V* is the volume, while *a* is the largest diameter on US, and *b* and *c* are the other two perpendicular diameters). Chest CT was used to detect distant metastases.

### RFA procedure

RFA was performed by experienced US physicians with more than 5 years of experience in thyroid US and interventional US. A bipolar RFA generator (CelonLabPOWER, Olympus Surgical Technologies Europe) and an 18-gauge bipolar RF applicator with a 0.9-cm active tip (CelonProSurge micro 100-T09, Olympus Surgical Technologies Europe) were used.

Patients underwent RFA with local anesthesia in an outpatient setting. The target tumor and its adjacent critical structures (such as the trachea, vessels, esophagus, and recurrent laryngeal nerves) were carefully evaluated under US and Doppler US to design the best insertion method. The ablation procedures were performed using the hydrodissection technique, moving-shot technique, and enlarged ablation technique (5). Normal saline was used as the isolating fluid. It was injected using a 23-gauge needle to establish a minimum distance of at least 1 cm between the target tumor and critical structures to prevent thermal injury (9). Under US guidance, the electrode was percutaneously inserted through the normal parenchyma and positioned within the deepest and most distal portion of the target tumor. During RFA, the target tumor was virtually divided into multiple small ablation units, and the electrode was moved unit-by-unit to allow the entire tumor to be treated. The physician enlarged the entire area, which exceeded the tumor edge (≥3 mm) to prevent marginal residue (18). The ablation was terminated when all units of the tumor had changed to hyperechoic areas. Contrast-enhanced ultrasound (CEUS) was performed immediately after ablation to evaluate the ablation area. If any enhancement was observed, a complementary ablation was performed. During RFA, special attention was given to the preservation of critical cervical structures to prevent significant complications. After RFA, each patient was observed for 1 to 2 h in the hospital while any complications occurring during and immediately after ablation were carefully evaluated.

### Post-ablation assessment

Patients were followed up at 1, 3, 6, 12, 18 months, and every 12 months thereafter. Laboratory tests, US, and CEUS were performed at each follow-up evaluation, and chest CT was performed 1 or 2 years to monitor for distant metastasis. US and CEUS were performed at each follow-up visit by physicians with 5 years of experience in thyroid US. Suspicious cervical LN (with at least one of the suspicious features, such as microcalcifications, partially cystic appearance, increased vascularization, and hypo-echogenicity) or newly developed tumor (ACR TI-RADS 4 or TI-TADS 5) was submitted to biopsy. The VRR was calculated using the following equation: VRR = ([initial volume − final volume] × 100)/initial volume.

### Clinical end points and definitions

The primary end points were local tumor progression (LTP), which was classified as follows (19): (1) cervical LNM confirmed by FNA or CNB, and (2) newly found PTC, defined as a new tumor identified in the lobe distant from the site of the ablated tumor, confirmed by FNA or CNB. Distant metastasis was confirmed by chest CT or surgical pathology. The treatment of LTP was managed by the physician and patient in consultation based on the patient’s circumstances and preferences.

The secondary end points included VRR, tumor complete disappearance, complications, and delayed surgery (19). Tumor complete disappearance was defined as follows: (1) the ablated tumor completely disappeared on US, or (2) the ablated tumor appearing scar-like on US with an absence of enhancements on both arterial and venous phases on CEUS. Complications during the ablation or follow-up were assessed (19). Delayed surgery was defined as patients who underwent surgery due to disease progression or anxiety during the follow-up (20, 21).

### Statistical analysis

Statistical analysis was performed using the SPSS statistical software (Version 25.0). Normally distributed continuous data were presented as mean ± SD (range) and non-normally continuous data were presented as median with interquartile range (IQR). Categorical data were expressed as numbers with percentages. The Wilcoxon signed-rank tests were used to compare the initial volume with post-ablation volume as each follow-up visit. A difference with *p* < 0.05 was considered statistically significant.

## Results

Clinical characteristics of patients are shown in **Table 1**. A total of 32 patients (25 women and 7 men) were included in this study. The mean age of the patients was 50.2 ± 10.0 years. The largest diameter (median with IQRs) of tumors was 5.0 (5.0, 8.0) mm, and the volume was 58.9 (33.5, 175.9) mm^3^. Two patients had hypothyroidism before RFA (6.3%). The time interval between lobectomy and recurrence was 37.0 (19.0, 84.0) months.

**Table 1:** Clinical characteristics.

Characteristics	Data
Age, years	50.2 ± 10.0
Sex	
Female	25 (78.1)
Male	7 (21.9)
Time interval between initial lobectomy and recurrence, months	37.0 (19.0, 84.0)
Location	
Right lobe	18 (56.2)
Left lobe	14 (43.8)
Largest diameter, mm	5.0 (5.0, 8.0)
Tumor volume, mm^3^	58.9 (33.5, 175.9)
Follow-up period, months	54.4 ± 24.3
Hypothyroidism before RFA	2 (6.3)

Data are expressed as mean ± SD or median (IQR) or frequency (percentage).

### Primary end points

The primary end points are shown in **Table 2**. During a mean follow-up of 54.4 ± 24.3 months, the incidence of LTP was 6.3% (2/32). No newly identified tumors or distant metastases were observed. Two patients developed cervical LNM (6.3%). One patient with central LNM chose active surveillance, and the volume remained stable throughout the 56-month follow-up. The other patient with lateral LNM underwent additional RFA. The metastatic lesion was successfully ablated and had completely disappeared at 12 months after additional RFA.

**Table 2:** Clinical characteristics and outcomes of two patients who developed lymph node metastasis after ablation.

Patients	Before RFA			After RFA		
	Sex/Age	Initial surgery	Recurrent tumor location	Metastatic lymph node location	Management	Outcomes
1	F/36	Left lobectomy	Right lobe	Right/level VI	Active surveillance	Stable volume
2	F/43	Left lobectomy	Right lobe	Right/level IV	Additional ablation	Complete disappearance

### The secondary end points

All the patients underwent RFA successfully. The power, RFA procedure time, and energy were 5.2 ± 1.2 W, 134.4 ± 78.1 s, and 619.4 ± 358.5 J, respectively.

**Figure 2:**
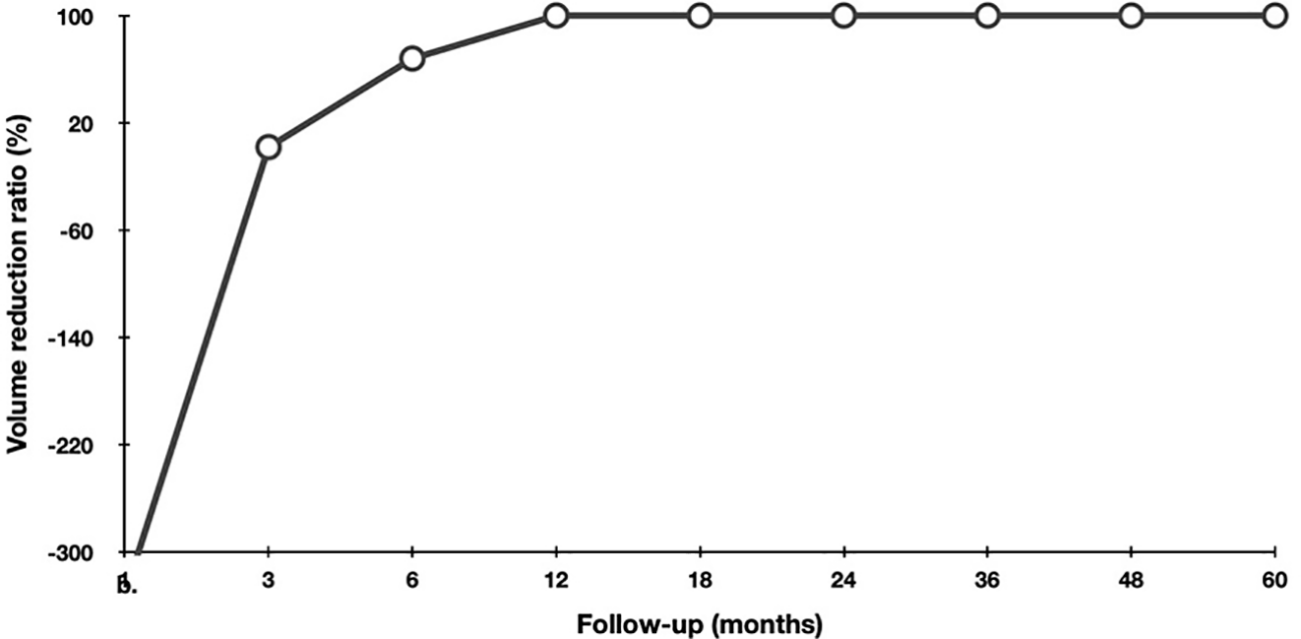
Changes of VRR during the follow-up.

**Figure 3:**
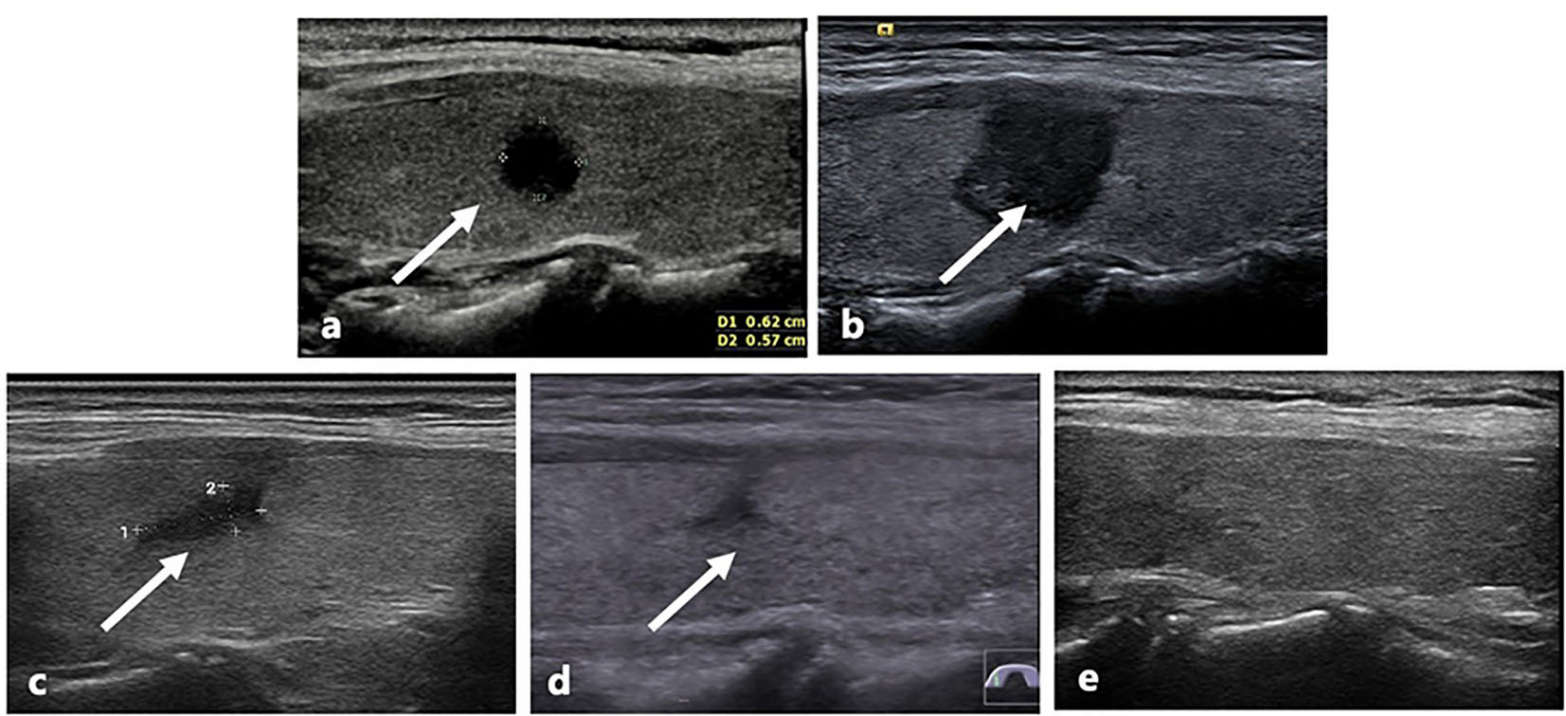
US image of a patient with recurrent tumor in the left lobe after lobectomy. **(a)** Before ablation, a recurrent tumor confirmed PTMC located in the left thyroid lobe (arrow) with an initial volume of 94.3 mm^3^. **(b)** At 1 month after RFA, the volume of the ablated area was 622.0 mm^3^ (arrow). **(c)** At 3 months after ablation, the volume was 113.1 mm^3^ (arrow). **(d)** At 6 months after ablation, the ablation area was 31.4 mm^3^ (arrow). **(e)** At 24 months after ablation, the ablated tumor disappeared.

During the follow-up, the initial volume of the tumor was 58.9 (33.5,175.9) mm^3^, which had decreased significantly to 0 (0, 0) mm^3^ (*p* < 0.001). The changes of volume and VRR at each follow-up point are shown in **Table 3**. Owing to the enlarged ablation, the volume measured at 1-month post-ablation was significantly larger than the initial volume (*p* < 0.001). After 6 months, the volume gradually decreased. At the last follow-up visit, the median VRR was 100% (**Figure 2**). The rate of tumor complete disappearance was 90.4% (29/32) (**Figure 3**).

**Table 3:** Changes of the volume and VRR after ablation.

Time	Volume (mm^3^)	VRR (%)	*p*-value (vs. initial volume)
Initial	58.9 (33.5, 175.9)	–	
Immediately after ablation	654.5 (357.3, 970.7)	–	<0.001
1 month	256.6 (198.9, 590.6)	−332.0 (−643.7, −129.1)	<0.001
3 months	119.4 (37.3, 219.5)	1.8 (−175.9, 34.5)	0.244
6 months	25.1 (0, 59.7)	67.9 (37.0, 100.0)	0.001
12 months	0 (0, 9.42)	100.0 (98.2, 100.0)	<0.001
18 months	0 (0, 0)	100.0 (100, 100)	<0.001
24 months	0 (0, 0)	100.0 (100, 100)	<0.001
36 months	0 (0, 0)	100.0 (100, 100)	<0.001
48 months	0 (0, 0)	100.0 (100, 100)	<0.001

Data are expressed as median with interquartile ranges.

VRR, volume reduction rate.

All the patients were tolerable to RFA procedure. The overall complication rate was 3.1% (1/32). One patient experienced voice change and resolved spontaneously within 1 week. Adverse effects, such as discomfort and mild pain, occurred in five patients, which gradually resolved within 1–3 days. No patient experienced hypothyroidism after RFA, and the changes of fT3, fT4, and TSH before and at the last-follow-up are shown in **Table 4**. Because of anxiety, one patient underwent total thyroidectomy with central compartment neck dissection 2 months post-ablation. RFA did not affect the course of the surgical procedure, and the pathological results showed nodular goiter, fibrous proliferation, and coagulative necrosis with lymphocyte infiltration. No residual cancer cells, occult tumors, or cervical LNMs were found on the surgical histopathology.

**Table 4:** The changes of fT3, fT4, and TSH before RFA and at the last follow-up.

Characteristics	Baseline	At the last follow-up	*p*-value
fT3	4.9 ± 0.9	5.1 ± 0.6	0.815
fT4	18.4 ± 4.3	20.2 ± 3.6	0.847
TSH	1.0 ± 1.1	0.4 ± 0.5	0.231

fT3, free triiodothyronine; normal range, 2.76–6.30 pmol/L.

fT4 free thyroxine; normal range, 10.42–24.32 pmol/L.

TSH, thyroid-stimulating hormone; normal range, 0.23–5.50 mU/L.

## Discussion

This study evaluated the clinical outcomes of RFA for recurrent contralateral tumors after initial surgery for PTC. The results showed that after a mean follow-up period of 54.4 ± 24.3 months, two patients developed cervical LNM. The overall rate of LTP was 6.3%. No newly identified tumors or distant metastases were observed. At the last follow-up, the median VRR was 100%, and 90.4% of the tumors had completely disappeared. No major complications or sequelae occurred after RFA. Moreover, thyroid function was well-maintained, and no patient experienced hypothyroidism following RFA.

Thyroid lobectomy has been recommended as the first-line surgical option for low-risk PTC (3). However, loco-regional recurrence can occur in the cervical LNs (1.2%–9.4%) and the remnant contralateral lobe (0.5%–6.5%) following lobectomy (22). Repeat surgery is the standard treatment. However, because of neck distortion, scar formation, and fibrous tissue adhesion resulting from the initial surgery, patients in the repeat surgery group had a higher incidence of hematoma formation (4.3% vs. 1.7%, *p* = 0.033) and transient voice change compared to those in the primary surgery group (5.5% vs. 2.5%) (23). RFA has been recommended as effective and safe alternatives to repeat surgery for recurrent thyroid cancers (4–11). Several studies have shown favorable outcomes of ablation for cervical LMM (14, 24–26). The pooled proportions of VRR and complete disappearance rate ranged from 88.4% to 93% and 67.9% to 82%, respectively, with a significant reduction in serum thyroglobulin levels (16, 17, 27). Additionally, comparative studies have reported comparable RFS and survival outcomes between thermal ablation and repeat surgery for cervical LMM (15, 28). However, information on the outcomes of RFA for contralateral tumors, developed after initial surgery for PTC, remains scarce.

This study enrolled 32 patients with unifocal recurrent contralateral tumors that developed after initial surgery for PTC. By one session ablation, the median VRR was 100%, and 90.4% of the tumors had completely disappeared. These results were consistent with those of previous meta-analyses on ablation for primary PTMC, which reported a pooled proportion of VRR of 92% and a complete disappearance rate ranging from 79% to 98.5% (20, 29, 30). Moreover, during 4-year follow-up, two patients experienced LNM, and one received additional RFA successfully. As a minimally invasive technique, RFA can achieve complete tumor destruction through the local application of extreme temperatures. The procedure can be performed effectively and repeatedly, with no increase in technical difficulties due to previous treatments (27). Moreover, because only the targeted tumor was treated during the ablation procedure, the incidence of permanent hypothyroidism was only 0.04% (1/2,245) (31). In this study, the thyroid function of patients was well-maintained, and no one experienced hypothyroidism following RFA.

Few complications occurred following RFA. The pooled proportion of overall and major complications of cervical LNM were 0%–5% and 0%–4.0%, respectively (16, 17, 21, 27). Voice changes were the most common complications, and most cases were transient and resolved spontaneously (16, 17, 27). In contrast, following repeat surgery, the rates of transient and permanent voice changes were 14.0% and 6.0%, respectively (23). This could be attributable to several reasons. First, the ablation procedure was performed by experienced physicians with knowledge of US-based neck structures and intervention skills. It was very important to minimize complications because the neck anatomical structures were distorted by initial surgery. Second, real-time US could allow the physician to monitor ablation procedure precisely. Third, several recommended ablation techniques were performed during the RFA procedure, including hydrodissection techniques and moving-shot techniques, which could also prevent thermal injury. Fourth, compared to repeat surgery, RFA requires local anesthesia in an outpatient setting, with a significantly shorter procedure time, thus leading to less trauma and a faster recovery (15, 28). Moreover, maintaining communication with patients during ablation was essential for monitoring voice changes. If voice hoarseness occurs, injection of a cold 5% dextrose solution could manage thermal nerve damage (32).

While RFA and other thermal ablation can be effective for the local tumor control, it cannot address small occult tumors or regional microscopic metastases that are undetected by imaging modalities (14, 33, 34). Therefore, repeat surgery is still the first-line treatment for contralateral tumor. The indications for RFA should be evaluated by a multidisciplinary team including members with expertise in interventional US to assess the demographic, clinical, histological, and imaging characteristics for appropriate selection. However, given that most survivors of low-risk PTC had a long life expectancy, a much safer and less invasive treatment choice may be more attractive for patients during the decision-making process. Therefore, for postoperative patients, who are unsuitable for or decline repeat surgery, RFA may be considered as a feasible and safe local treatment option for recurrent contralateral tumors after rigorous pre-ablation evaluation.

This study has several limitations. First, this was a retrospective single-arm study with inherent selection bias. Prospective controlled studies are needed to validate these findings. Second, the sample size of this study was relatively small. Third, the study population consists of carefully selected low-risk patients. Because most patients underwent their initial surgery several years prior, detailed information regarding the primary tumor was insufficient, which may have an impact on the prognosis. Therefore, the study population consists of carefully selected low-risk patients. All the patients underwent rigorous pre-ablation evaluation. Only patients with one contralateral PTMC tumor were involved in this study. The outcomes of RFA for other types of thyroid cancer or intermediate-/high-risk patients with PTC still need further study. Fourth, as all the patients underwent lobectomy only for initial PTC, the clinical value of serum thyroglobulin levels was limited. Therefore, serum thyroglobulin levels were not assessed in this study. Fifth, we did not compare repeat surgery/active surveillance and RFA for recurrent contralateral tumors.

In conclusion, RFA may be considered as a feasible and safe local treatment for recurrent contralateral tumors after the initial surgery for PTC in select patients who are not candidates for or who refuse repeat surgery.

## Data Availability

The datasets presented in this article are not readily available because Due to confidentiality and patient privacy, we cannot provide the original data. Requests to access the datasets should be directed to longjun531@163.com.
